# RNA-Seq Analysis to Identify Novel Roles of Scleraxis during Embryonic Mouse Heart Valve Remodeling

**DOI:** 10.1371/journal.pone.0101425

**Published:** 2014-07-01

**Authors:** Damien N. Barnette, Matthew VandeKopple, Yonggan Wu, David A. Willoughby, Joy Lincoln

**Affiliations:** 1 Molecular and Cellular Pharmacology Graduate Program, Leonard M. Miller School of Medicine, Miami, Florida, United States of America; 2 Center for Cardiovascular and Pulmonary Research and The Heart Center at Nationwide Children's Hospital Research Institute, Columbus, Ohio, United States of America; 3 Molecular, Cellular and Developmental Biology Graduate Program, The Ohio State University, Columbus, Ohio, United States of America; 4 Ocean Ridge Biosciences LLC, Palm Beach Gardens, Florida, United States of America; 5 Department of Pediatrics, The Ohio State University, Columbus, Ohio, United States of America; Northwestern University, United States of America

## Abstract

Heart valve disease affects up to 30% of the population and has been shown to have origins during embryonic development. Valvulogenesis begins with formation of endocardial cushions in the atrioventricular canal and outflow tract regions. Subsequently, endocardial cushions remodel, elongate and progressively form mature valve structures composed of a highly organized connective tissue that provides the necessary biomechanical function throughout life. While endocardial cushion formation has been well studied, the processes required for valve remodeling are less well understood. The transcription factor Scleraxis (Scx) is detected in mouse valves from E15.5 during initial stages of remodeling, and expression remains high until birth when formation of the highly organized mature structure is complete. Heart valves from *Scx^-/-^* mice are abnormally thick and develop fibrotic phenotypes similar to human disease by juvenile stages. These phenotypes begin around E15.5 and are associated with defects in connective tissue organization and valve interstitial cell differentiation. In order to understand the etiology of this phenotype, we analyzed the transcriptome of remodeling valves isolated from E15.5 *Scx^-/-^* embryos using RNA-seq. From this, we have identified a profile of protein and non-protein mRNAs that are dependent on Scx function and using bioinformatics we can predict the molecular functions and biological processes affected by these genes. These include processes and functions associated with gene regulation (methyltransferase activity, DNA binding, Notch signaling), vitamin A metabolism (retinoic acid biosynthesis) and cellular development (cell morphology, cell assembly and organization). In addition, several mRNAs are affected by alternative splicing events in the absence of *Scx*, suggesting additional roles in post-transcriptional modification. In summary, our findings have identified transcriptome profiles from abnormal heart valves isolated from E15.5 *Scx^-/-^* embryos that could be used in the future to understand mechanisms of heart valve disease in the human population.

## Introduction

Heart valves facilitate unidirectional blood flow during the cardiac cycle and this is largely achieved by highly organized layers of connective tissue that each offer distinct biomechanical properties to facilitate opening and closing of the valve leaflets or cusps [1], [2]. In healthy valves, connective tissue homeostasis is mediated by valve interstitial cells (VICs), which in turn are regulated by a monolayer of valve endothelial cells (VECs) that overly the valve surface. In contrast, diseased or dysfunctional valves from pediatric and adult patients are characterized by VIC disarray, VEC denudation and disorganization of the connective tissue leading to biomechanical insufficiency [3], [4]. At present, there is no effective treatment for valve disease and over 50,000 valve replacement surgeries are performed in the United States each year [5]. Many of these procedures using bioprosthetic or mechanical valves fail due to structural or thrombotic-related problems and therefore alternatives are needed [6]. In order to improve therapeutic strategies, it is imperative that we understand the underlying pathogenesis of diseased valves. Defects in embryonic valve development are responsible for congenital heart valve malformations, however there is increasing evidence to suggest that they also attribute to disease later in life [2].

Mature heart valve leaflets and supporting structures are largely derived from a population of mesenchyme precursor cells within endocardial cushions that form as a result of endothelial-to-mesenchyme transformation (EMT) in the atrioventricular canal and outflow tract regions during embryogenesis [4], [7]. Defects in generating this pool of valve precursor cells often results in embryonic lethality and therefore valvular phenotypes caused by severe endocardial cushion-related defects are not frequently observed in live births [8]. Once EMT is complete, endocardial cushions remodel and elongate to form primitive valve primordia [2]. During this time, valve precursor cells lose their mesenchymal phenotype and become activated VICs that mediate breakdown of the primitive extracellular matrix (ECM) within the endocardial cushion, and secrete specialized matrix components that will later form the mature valve structures [3]. In the mouse, valve remodeling begins around E15.5 and continues until post natal stages, when the VICs become quiescent and the organized ECM is established and maintained [2]. Compared to endocardial cushion formation, the process of valve remodeling is not well studied but thought to require tight regulation of complex signaling pathways and cellular processes. It is hypothesized that defects during this important stage of valvulogenesis may underlie valve defects observed in pediatric patients at birth, and in adults later in life.

Scleraxis (Scx) is a member of the basic helix-loop-helix family of transcription factors required for development of tissues of high mechanical demand including tendons and heart valves [9], [10]. We have previously shown that Scx is not expressed in developing heart valve structures during stages of endocardial cushion formation, but is first detected around E15.5 at the beginning of valve remodeling [9]. In embryos null for *Scx*, mesenchymal phenotypes are prolonged in valve precursor cells suggesting defects in VIC maturation. At birth, the valve leaflets are thickened and the ECM is highly unorganized, and by juvenile stages leaflets from *Scx^-/-^* mice are grossly malformed and display characteristics of pathological fibrosis including excess collagen deposition [9]. In more recent work, we have shown that Scx plays additional roles in regulating components of the valve ECM by promoting expression of proteoglycans. Furthermore, we identified increased *Scx* expression in thickened heart valves isolated from mouse models and patients of myxomatous mitral valve disease [11]; a disorder associated with valvular insufficiency caused by an abnormal abundance of proteoglycans. Together, these studies show that Scx is required during embryonic valve remodeling for formation of heart valve structures after birth. Despite this, the downstream targets and functional role(s) of Scx during this stage of valvulogenesis remain elusive.

To address this current deficit we took an RNA-seq approach to analyze the transcriptome of remodeling heart valves isolated from E15.5 *Scx^-/-^* embryos compared to wild type (*Scx^+/+^*) littermates. Using this approach we have identified previously unappreciated protein-coding and non-protein coding mRNAs that are differentially expressed in the absence of *Scx*. Based on our previous studies, we were surprised to see that biological processes and molecular functions associated with the valve ECM were not significantly altered in *Scx^-/-^* embryos at E15.5. However, we report enrichment of mRNAs associated with processes related to gene regulation (methyltransferase, DNA binding, nucleosomal binding, miRs, signaling) vitamin A metabolism (biosynthetic processes), and cellular development (cell assembly and organization). Furthermore, bioinformatics analysis revealed known and predicted novel upstream regulators of Scx in the valves at this time of embryonic development. In addition to differential gene expression changes, splicing index analysis identified several mRNAs affected by differential exon abundance in the absence of *Scx*. Together, these findings identify genes and hierarchical networks regulated by *Scx* in remodeling heart valves and provide insights into molecular and cellular processes that when abrogated, could underlie disease.

## Materials and Methods

### Murine Tissue Collection

*Scx^-/-^* and *Scx^+/+^* littermate mice were generated as previously described [9] and collected at embryonic day (E) 15.5, counting day E0.5 by evidence of a copulation plug. Atrioventricular canal (AVC) regions containing mitral, tricuspid and aortic valves from *Scx^+/+^* (n = 3) and *Scx^-/-^* (n = 3) embryos were dissected from hearts with minimal myocardial contamination and RNA was extracted using Trizol reagent (Invitrogen). Alternatively, whole hearts were collected and prepared as described below for immunohistochemical analysis. This study was carried out in strict accordance with the recommendations in the Guide for the Care and Use of Laboratory Animals of the National Institutes of Health. The mouse protocol (AR11-00076) was approved by the IACUC Committee at Nationwide Children's Hospital.

### RNA preparation and processing

Total RNA samples were sent to Ocean Ridge Biosciences LLC (Palm Beach Gardens, FL) for quality control analyses and processing. RNA concentration was determined by ribogreen fluorometry, and RNA integrity and purity assessed using agarose gel electrophoresis. All samples had RNA Integrity of “1”, indicating intact RNA with strong ribosomal banding. First- and second-strand cDNA was synthesized from purified RNA and library constructed.

### RNA sequencing and data processing

The cDNA library for each sample was sequenced using the Illumina HiSeq 2000 instrument and sequencing by synthesis (SBS) technology (Illumina, San Diego, CA, USA). Tophat 1.4.1 software was used to align the library reads to the UCSC Mouse (mm9) reference genome (>75% efficiency), and annotated using Samtools v0.1.18. EasyRNASeq version 1.6 was used to count reads mapping within Ensembl version 66 exons, and calculate normalized counts for each gene. Raw count files were annotated using data from Ensembl Mouse version 66. Reads per kilo-base per million (RPKM) values were calculated using easyRNASeq output, and automatically processed using Perl version 5.10.1. RPKM values were filtered to retain genes with a minimum of ∼50 mapped reads in one or more samples. The threshold of 50 mapped reads is considered the Reliable Quantification Threshold, as RPKM values for a gene represented by 50 reads should be reproducible in technical replicates. To avoid reporting large fold changes due to random variation of counts from low abundance mRNA, RPKM values equivalent to a count of ≤10 reads per gene were replaced with the average RPKM value equivalent to 10 reads/gene across all the samples in the experiment. One-way ANOVA was performed and fold changes were calculated using R version 3.0 statistical computing software. If the mean of both groups considered in a fold-change comparison were below the Reliable Detection Threshold (50 reads/gene), “NA” was reported. Significant fold changes were considered with p-value <0.05. Raw data files of the RNA-seq analysis can be accessed through GEO (Gene Expression Omnibus) Data Sets, accession GSE57423.

### Venn Diagram

All detectable genes in *Scx^-/-^* and *Scx^+/+^* samples were selected for representation in a Venn diagram. Genes were considered ‘undetectable’ if the RPKM was below the Detection Threshold for the corresponding sample. If at least one of the gene reads from a triplicate sample set was proven undetectable while all gene reads in the comparative sample set was proven detectable, the gene was considered uniquely expressed in that sample. If the gene was read from both triplicate sample sets had detectable RPKM values above the Detection Threshold, the gene was considered common amongst sample groups. Genes with at least one triplicate below the Detection Threshold in both sample sets are not represented in the Venn diagram.

### Clustering analysis

Genes corresponding to differentially expressed transcript clusters were selected for display in hierarchical clustering, with threshold criteria of p<0.05 in a one-way ANOVA analysis. The 862 differentially expressed genes were clustered using Cluster 3.0 software. The log2-transformed data were pre-processed by median centering, and then hierarchically clustered using centered correlation as the similarity metric and average linkage as the clustering method.

### Alternative Splicing Indexes

The normalized counts of sequence reads (RPKM) mapped to annotated UCSC exons were determined using easyRNASeq software. The annotations for each gene were added from Ensembl BioMart. The exon-level RPKM values were filtered in two steps. First, exons were discarded if their corresponding genes did not reach the Reliable Quantification Threshold (∼50 reads/gene) in at least one sample. Second, exons were discarded if the exons were not detected (at least one read/exon) in at least one sample. Prior to calculating Splicing Indexes, the exon data was adjusted such that RPKM values of ≤1 read/exon were replaced with the RPKM that was equivalent to 1 read/exon, as calculated from an average of all samples in the data set. The Splicing Indexes were calculated based on the formula: exon RPKM/gene RPKM. The Splicing Index value for a given exon and sample were replaced with “NA” if the corresponding gene count was not reliably detected (<50 reads/gene). ANOVA and Tukey test were performed to determine statistically significant differences in *Scx^-/-^* vs. *Scx^+/+^* samples, and significance of “NA” was reported for an exon if the Splicing Index of one or more samples was set to “NA” due to low or absent gene level expression.

### Pathway Analysis

Identified differentially expressed genes were further analyzed for the inclusion in gene ontology and pathway analysis in order to determine the distribution of genes amongst functional biological processes. WebGestalt software (Vanderbilt University) was utilized for a statistics-based pathway analysis to compare the relative distribution of genes that met specific significance criteria to the distribution of all detectable genes. Statistical significance is based on an adjusted p-value <0.05 for enrichment of genes meeting the selection criteria, relative to the reference genes in specific pathways. The WebGestalt software was used to query three pathway databases including KEGG, Wiki Pathways, and GO Pathways. Additional analyses were performed using Ingenuity Pathway Analysis software (IPA, Ingenuity Systems, Redwood City, CA, USA). The annotated genes were grouped into networks, functions, and/or canonical pathways. The txt. files with gene IDs, fold change expression, and p-values were uploaded in the software, and genes were mapped into corresponding gene objects in the Ingenuity Knowledge Base (IKB). Genes with fold change >1.5 and p value <0.05 were used to generate a network of focus genes into global molecular networks and predicted upstream signaling pathways. Fisher's exact test was used to identify the most significantly (p<0.05) altered biological functions and/or diseases within the dataset.

### Immunohistochemistry

Whole hearts were harvested from E15.5 *Scx^-/-^* and *Scx^+/+^* embryos and fixed in 4% paraformaldehyde (PFA) overnight and processed for paraffin embedding as described [9]. 7 µm tissue sections were cut, deparaffinized and rehydrated through an ethanol gradient series and washed three times for ten minutes in 1xPBS containing 0.5% Triton-X. Tissue sections were then incubated in blocking solution for one hour at room temperature as described [12], followed by overnight incubation with primary antibodies against Fibromodulin (Fmod) (Santa Cruz sc-33772, 1∶50), Heparin Sulfate Proteoglycan 2 (Hspg2) (Santa Cruz sc-25848, 1∶100) and Collagen type 4 (Col4) (Santa Cruz sc-9301, 1∶50). Tissue sections were then washed in 1xPBS and incubated with appropriate Alexa-fluor-488 secondary antibodies for one hour at room temperature in the dark. Following several washes with 1x PBS, tissue sections were mounted with Vectashield containing DAPI nuclei stain.

### Rat Valve Interstitial Cell Isolation

Valve interstitial cells (VICs) were isolated from three, Sprague-Dawley rats and cultured as previously described [13]. This study was carried out in strict accordance with the recommendations in the Guide for the Care and Use of Laboratory Animals of the National Institutes of Health. The rodent protocol (AR14-00017) was approved by the IACUC Committee at Nationwide Children's Hospital).

### Scleraxis Gain of Function Assays

Passage 1-3 rat VICs were plated on 4kPa collagen-coated polyacrylamide substrates at a density of 200,000 cells/well (6 well). For Scx overexpression assays, rat VICs were equilibrated in serum-free rat VIC media for 24 h prior to treatment. Cells were then treated with 3×10∧7 AdV-Scx or AdV-GFP in serum-free VIC media for 24 h and RNA was isolated.

### Quantitative PCR

RNA was extracted from treated rat VICs using standard Trizol protocols (Invitrogen). 200 ng of RNA was synthesized into cDNA using the RNA to cDNA synthesis kit (Applied Biosystems) according to the manufacturer's protocol. cDNA was diluted 1∶5, and quantitative real-time PCR was performed using 5 µL cDNA and 10 µl Taqman Master mix (Applied Biosystems) with 0.5 µl each primer (at 20 pmol/ µl) as described [12] with the following TaqMan assays (Applied Biosystems): *Scx* (Rn1504576_m1), *Actn4* (Mm00502489_m1), *Dot1L* (Rn01535507_m1), *Aldh1a1* (Rn00755484_m1) and *18s* (4352930). Cycle counts for each target gene were normalized to *18s* expression, and significant differences in gene expression were reported as a fold change compared to respective controls.

## Results

### Pairwise and clustering analysis distinguishes E15.5 *Scx^-/-^* atrioventricular canal regions from controls

**Figure 1:**
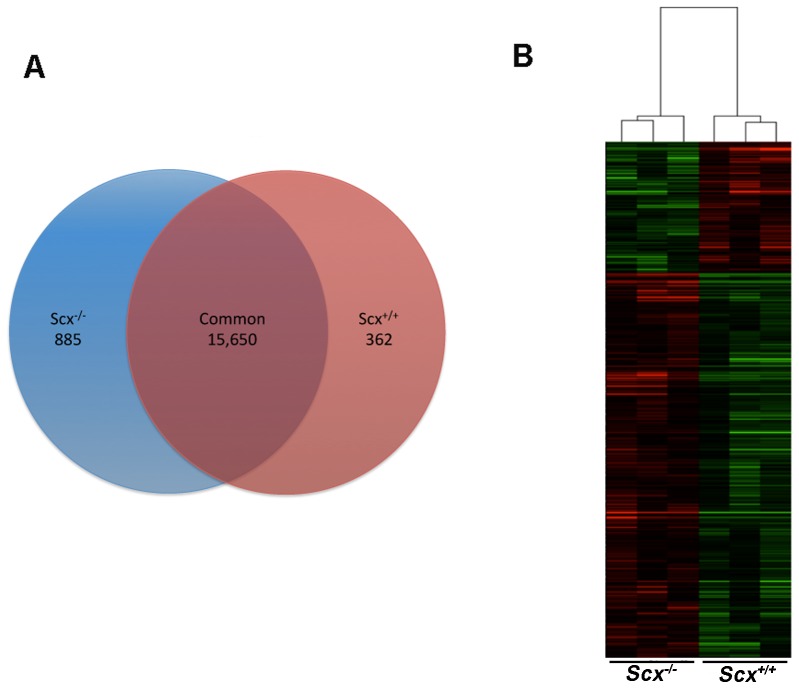
Loss of *Scx* function in remodeling murine heart valves at E15.5 leads to distinct transcriptome profiles. (A) Venn diagram to show the number of detectable protein-coding and non-protein coding mRNAs that were unique and common to *Scx^+/+^* and *Scx^-/-^* samples. (B) Heat map to show hierarchical clustering of differentially expressed genes (>1.5-fold change, p<0.05) in *Scx^+/+^* and *Scx^-/-^* samples.

As we have previously shown that Scx is highly expressed in heart valves from E15.5 and required for formation of valvular structures [9], examining differential gene expression changes in null mice could provide insights into the potential function of Scx during valve remodeling. To do this, we performed global transcriptome analysis in atrioventricular canal regions containing mitral, tricuspid and aortic valves, isolated from E15.5 *Scx^-/-^* (n = 3) and *Scx^+/+^* (n = 3) hearts. Samples were subject to RNA sequencing (RNA-seq) using Illumina HiSeq 2000 following confirmation of a 1523.23 ± 58.68 fold decrease in Exon 1 expression in *Scx^-/-^* samples compared to controls, as previously described [10]. Annotation from Ensembl and Reliable Quantification Threshold settings (50 RPKM) resulted in a total of 18,810 detectable genes. Pairwise comparisons between *Scx^-/-^* and *Scx^+/+^* sample groups were made and 362 mRNAs were found to be uniquely expressed in *Scx^-/-^* samples, 885 in *Scx^+/+^* control samples, and 15,650 genes were commonly expressed in both sample sets (Figure 1A). 2,798 genes were categorized as ‘undetected’ after Detection Threshold criteria. These observations suggest that a total of 1,247 genes are regulated in a Scx-dependent manner in remodeling heart valve at E15.5. The top 25 most differentially expressed protein-coding mRNAs in *Scx^-/-^* samples are indicated in Table 1, and affected non-protein-coding genes are shown in Table S1.

**Table 1:** Top 25 most differentially expressed protein-coding mRNAs (>1.5-fold change, p<0.05) in *Scx^-/-^* atrioventricular canal samples compared to *Scx^+/+^* controls.

Gene	Description	Fold Change	p-value
*Ly6i*	lymphocyte antigen 6 complex, locus I	11.01	7.98E-03
*Cst9*	cystatin 9	8.01	2.76E-04
*Mlana*	melan-A	6.78	1.13E-02
*Dnajb7*	DnaJ (Hsp40) homolog, subfamily B, member 7	5.78	2.16E-02
*Akr1b7*	aldo-keto reductase family 1, member B7	5.76	1.46E-02
*Glrp1*	glutamine repeat protein 1	5.69	3.09E-02
*Rnase1*	ribonuclease, RNase A family, 1 (pancreatic)	5.10	2.60E-03
*Pacrg*	PARK2 co-regulated	4.62	1.05E-02
*Gatsl3*	GATS protein-like 3	3.64	1.02E-02
*Rpusd1*	RNA pseudouridylate synthase domain containing 1	3.61	6.27E-03
*Rad54b*	RAD54 homolog B (S. cerevisiae)	2.35	4.10E-02
*Ftsj2*	FtsJ homolog 2 (E. coli)	2.35	1.67E-02
*Nppc*	natriuretic peptide type C	3.10	4.88E-02
*Pvalb*	parvalbumin	3.10	3.85E-02
*Apobec3*	apolipoprotein B mRNA editing enzyme, catalytic polypeptide 3	2.96	2.58E-02
*Mnda*	myeloid cell nuclear differentiation antigen	2.89	3.70E-02
*C1qtnf2*	C1q and tumor necrosis factor related protein 2	2.69	2.51E-02
*Aard*	alanine and arginine rich domain containing protein	2.68	3.28E-02
*Trmt61a*	tRNA methyltransferase 61 homolog A (S. cerevisiae)	2.56	1.60E-02
*Parvb*	parvin, beta	0.40	1.81E-02
*Shank3*	SH3/ankyrin domain gene 3	0.41	7.25E-03
*Megf10*	multiple EGF-like-domains 10	0.42	2.93E-02
*Hlx*	H2.0-like homeobox	0.42	2.25E-02
*Cml1*	camello-like 1	0.29	1.67E-02
*B3galt4*	UDP-Gal:betaGlcNAc beta 1,3-galactosyltransferase, polypeptide 4	0.31	3.43E-02

To further examine changes in gene expression, one-way ANOVA analysis was performed to compare significant differences in the core 18,810 detectable gene expression profiles in *Scx^-/-^* samples and *Scx^+/+^* controls. Of these, a total of 862 genes were differently expressed with a p-value of <0.05. 645 genes were upregulated, while 217 genes were downregulated in *Scx^-/-^* samples compared to controls. To visually represent commonality or variance in the pattern of the 862 differentially expressed genes between the two sample groups, hierarchical clustering and heat map analyses were performed. As shown in Figure 1B, *Scx^-/-^* samples clustered indifferently from controls, suggesting similar gene expression profiles.

### Scleraxis loss and gain of function leads to differential changes in expression of candidate mRNAs

**Figure 2:**
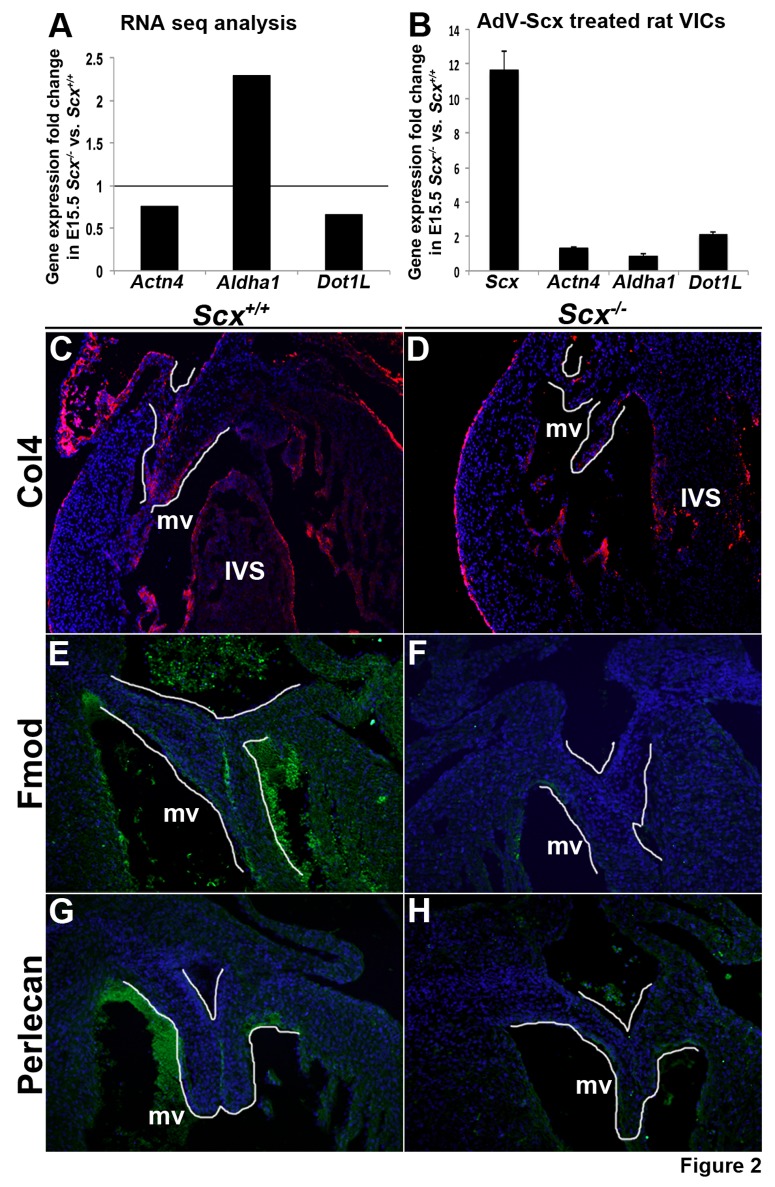
Validation of RNA-seq analysis. (A) Fold changes in gene expression of *Actn4, Aldha1* and *Dot1L* based on RNA-seq analysis. Grey line indicates normalized gene expression levels in *Scx^+/+^* controls (set at 1). (B) qPCR to show fold changes in *Scx, Actn4, Aldha1* and *Dot1L* expression in rat VICs infected with an adenovirus overexpressing Scx (AdV-Scx) compared to AdV-GFP infected controls. *, p<0.05, n = 3. Grey line indicates normalized gene expression levels in AdV-GFP treated rat VICs (set at 1). (C-H) Fluorescent immunohistochemistry to detect Type IV Collagen (Col4) (C, D at 10x magnification), Fibromodulin (Fmod) (E, F at 20x magnification) and Heparin sulfate proteoglycan 2 (Hspg2) (G, H at 20x magnification) in mitral valves (highlighted by white line) from E15.5 *Scx^+/+^* controls (C, E, G) and *Scx^-/-^* (D, F, H) embryos. Note reduced expression in tissue sections from *Scx^-/-^* embryos. mv, mitral valve; IVS, interventricular septum.

RNA-seq analysis revealed that 862 mRNAs were differentially expressed in atrioventricular canal regions from E15.5 *Scx^-/-^* mice. To validate some of these as potential direct or indirect targets of Scx, we performed gain of function experiments in rat valve interstitial cells (rat VICs) by infecting with an adenovirus containing full length Scx (AdV-Scx) or AdV-GFP that served as a control [11]. Following infection, changes in expression of *Actn4, Aldha1* and *Dot1L* were examined by qPCR. Figure 2A indicates fold changes in expression of these mRNAs from RNA-seq findings, while Figure 2B demonstrates that *Scx* overexpression in rat VICs leads to significant increases in *Actn4* and *Dot1L*. *Aldha1* expression was unchanged in response to Scx gain of function, which may reflect phenotypic differences between embryonic murine atrioventricular canal regions and young rat VIC cultures.

In addition to qPCR validation, immunostaining was performed to confirm differential changes in expression of Collagen 4 (Col4) (*Col4a1* 0.67-fold, p = 0.00053*; Col4a2* 0.68-fold, p = 0.0007) (Figure 2C, D), Fibromodulin (Fmod) (0.58-fold, p = 0.0049) (Figure 2E, F), and Heparin sulfate proteoglycan 2 (Hspg2) (0.55-fold, p = 0.0028) (Figure 2G, H) on tissue sections of mitral valves from E15.5 *Scx^-/-^* embryos. Consistent with RNA-seq analysis (indicated in parentheses), immunostaining shows decreased protein expression levels of these targets in mitral valves from *Scx^-/-^* embryos (Figures 2D, F, H) compared to *Scx^+/+^* controls (Figures 2 C, E, G).

### Pathway analysis reveals enrichment of differentially expressed mRNAs associated with gene regulation, vitamin A metabolism and cellular development are significantly altered in E15.5 atrioventricular canal regions from *Scx^-/-^* samples

**Figure 3:**
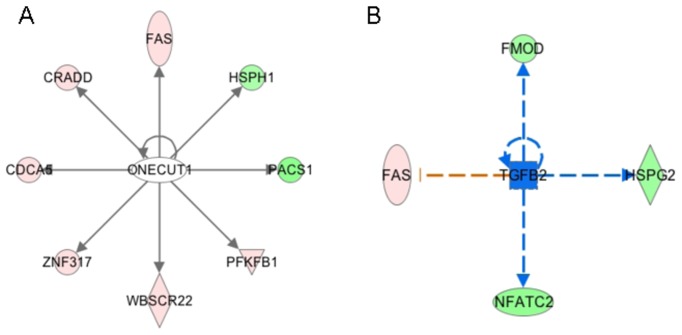
Predicted upstream regulators of Scx in remodeling heart valves. Ingenuity software analysis of differential gene expression changes (>1.5-fold change, p<0.05) in *Scx^-/-^* samples to predict upstream regulators of Scx. Based on Ingenuity prediction software, the molecule shapes indicate: ellipse = transcriptional regulators, triangle = kinase, circle = other, square = cytokine, diamond = enzyme. For molecular shape colors: red = upregulated mRNAs, green = downregulated mRNAs, white = no part of dataset, blue = canonical pathway. For lines, solid grey = effect not predicted, dashed line = indirect interaction (blue = positive indirect interaction, orange = negative indirect interaction).

To determine the biological processes and molecular functions altered by the loss of *Scx* in E15.5 heart valves, pathway analysis was performed. Of the 862 differentially expressed genes that met criteria threshold (p-value <0.05), 300 showed a significant fold change >1.5. Of these 300,238 (157 increased, 81 decreased) had annotated Entrez identification numbers and were therefore used for subsequent pathway analysis using Gene Ontology (GO), KEGG, Wiki and Ingenuity IPA softwares. We found that differentially expressed mRNAs in *Scx^-/-^* versus *Scx^+/+^* samples are largely associated with mechanisms related to gene regulation, vitamin A metabolism and cellular development processes (Table 2). These include mRNAs associated with methyltransferases, regulatory DNA binding, and Notch signaling pathways, all of which have been shown to regulate expression and function of target genes. In addition, predicted changes in 9-cis-retinoic acid-, vitamin A- and retinoic acid-biosynthesis and metabolic processes were observed. Significant changes in genes associated with cell development, cell morphology, cellular assembly and organization, and cell death and survival suggest an additional role for Scx in remodeling heart valves. In addition to pathway analysis, Ingenuity IPA software was used to predict upstream regulators of Scx in this system. Based on differential gene expression changes (fold change >1.5, p-value <0.05) 132 targets were predicted as upstream regulators of *Scx*, including the known regulator Tgfβ, [11], [14], [15] which was ranked number 2 based on p-value (Figure 3A), as well as the number 1 ranked Onecut1 (Figure 3B), a member of the Cut homeobox family of transcription factors involved in DNA binding. Together these bioinformatic approaches have revealed previously unappreciated networks and processes that are potentially mediated by Scx in remodeling heart valves.

**Table 2:** Bioinformatics pathway analysis to predict molecular functions and biological processes significantly affected by the loss of Scx in heart valves at E15.5.

Molecular function/Biological process	Bioinformatics Source and Associated Pathway Number	p-value	Gene	Fold Change
Lysine N-methyltransferase activity	GO:0016278	0.0005	*Dot1l*	0.66
Histone lysine N-methyltransferase	GO:0018024	0.0005	*Mll2*	0.52
Protein lysine N-methyltransferase activity	GO:0016279	0.0005	*Wbp7*	0.54
Histone methyltransferase activity	GO:0042056	0.0009		
Protein methyltransferase activity	GO:0008276	0.0022		
N-methyltransferase activity	GO:0008170	0.0017		
S-adenosylmethionine-dependent methyltransferase activity	GO:0008757	0.0058		
Transcription regulatory region DNA binding	GO:0044212	0.0071	*Junb*	0.49
Regulatory region nucleic acid binding	GO:0001067	0.0071	*Hlx*	0.42
Regulatory region DNA binding	GO:0000975	0.0071	*Nfatc2*	0.53
			*Ncor2*	0.46
			*Mll2*	0.52
Notch binding	GO:0005112	0.0007	*Dll4*	0.61
			*Ncor2*	0.46
9-cis-retinoic acid biosynthesis process	GO:0042904	0.00006	*Aldh1a1*	2.29
9-cis-retinoic acid metabolic process	GO:0042905	0.00006	*Aldh1a2*	1.52
Vitamin A biosynthetic process	GO:0035238	0.00006		
Retinoic acid biosynthetic process	GO:0002138	0.00020		
Diterpenoid biosynthetic process	GO.0016102	0.00020		
Terpenoid biosynthetic process	GO:0016114	0.00040		
Fat-soluble vitamin biosynthetic process	GO:0042363	0.00009		
Retinoic acid metabolic process	GO:0042573	0.00130		
3-chloroallyl aldehyde dehydrogenase activity	GO:0004028	0.00040		
Retinal dehydrogenase activity	GO:0001758	0.00040		
Nucleosomal DNA binding	GO:0031492	0.0006	*Hmgn5*	1.60
			*Hmgn3*	1.61
Secondary active transmembrane transporter activity	GO:0015291	0.0122	*AI317395*	0.64
			*Slc9a8*	0.66
			*Slc16a4*	0.54
Cysteine and methionine metabolism	KEGG:00270	0.0027	*Mpst*	1.74
			*Trdmt1*	1.59
			*Cdo1*	1.64
Pentose and glucuronate interconversions	KEGG:00040	0.0079	*Akr1b7*	5.76
			*Aldh1a1*	2.29
Notch Signaling Pathway	KEGG:04330	0.0090	*Dll4*	0.61
			*Ncor2*	0.46
			*Maml1*	0.64
Notch Signaling Pathway	Wiki	0.0010	*Dll4*	0.61
			*Ncor2*	0.46
			*Maml1*	0.64
	Wiki	0.0146	*Dll4*	0.61
Delta-Notch Signaling Pathway			*Ncor2*	0.46
			*Maml1*	0.64
Cellular Development	Ingenuity: IPA	3.26E-04	*Fam20c*	0.57
			*Fas*	1.76
			*Gpc4*	0.52
			*Hspg2*	0.55
			*Junb*	0.56
			*Lrp5*	0.49
			*Maml1*	0.56
			*Ncor2*	0.64
			*Nfatc2*	0.46
			*Nod1*	0.53
			*Nppc*	0.51
			*Nucb2*	3.10
Cell Morphology	Ingenuity: IPA	4.52E-04	*Efna5*	0.55
			*Fas*	1.76
Cellular Assembly and Organization	Ingenuity: IPA	4.52E-04	*Fas*	1.76
			*Plec*	0.66
Cellular Compromise	Ingenuity: IPA	4.52E-04	*Fas*	1.76
			*Plec*	0.66
Cell Death and Survival	Ingenuity: IPA	1.25E-03	*Maml1*	0.64
			*Plec*	0.66

(GO, Gene Ontology).

### Exon abundance is significantly altered in the absence of *Scx*

**Figure 4:**
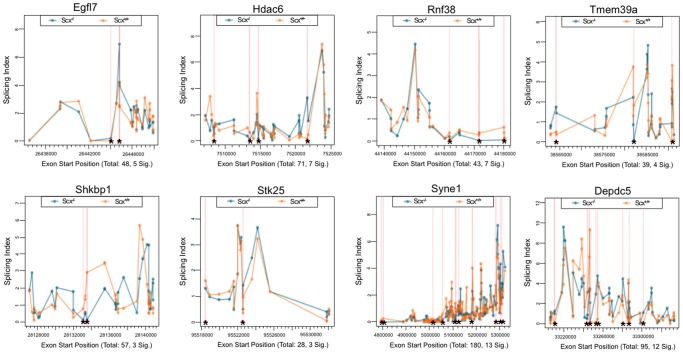
Exon-level splicing indices of mRNAs affected by alternative splicing events in *Scx^-/-^* samples at E15.5. Splicing indices of the top 8 mRNAs affected by changes in exon abundance (at least 10 exons) in the absence of Scx. * indicate significant differences in exon abundance (p<0.05).

To determine alterations in the abundance of individual exons of detectable genes in *Scx^-/-^* samples, exon-level expression profiling was performed using easyRNASeq. A total of 99 protein-coding genes were significantly altered by splicing (p<0.05), and these are shown in Table S2. Figure 4 plots the splicing index of each exon (at least 10) for the top 8 most significantly spliced genes in our samples and these include *Egfl7*, *Hdac6* and *Rnf38.* As indicated by the asterisks, *Scx^-/-^* samples show distinct exon-specific expression profiles compared to controls, and suggest a role for Scx in post-transcriptional events during valve remodeling.

## Discussion

To date, studies defining the role of Scx have focused on connective tissues of high mechanical demand including tendons, cardiac fibroblasts and heart valves [9], [11], [14]–[16]. Interestingly, defects in ECM organization and cell differentiation are commonly observed in all these affected structures in null mice [9], [11], [14]–[16], however the underlying causes are not understood but likely conserved. In heart valves, these phenotypes begin in the developing embryo and by birth, valves are abnormally thick and progressively worsen over time [9]. In order to define the roles that Scx plays in connective tissue systems and identify how loss of function gives rise to valve anomalies, RNA-seq was performed in valvular regions isolated from E15.5 *Scx^-/-^* embryos. Using this approach, we have identified previously unappreciated genes that are dependent upon Scx function and predicted novel upstream regulators. In addition, bioinformatics has unveiled potential new roles for Scx in remodeling heart valves associated with gene regulation, vitamin A metabolism and cell development processes. Together, these findings provide new insights into the mechanisms of Scx function in remodeling heart valves that could have implications in disease pathogenesis.

Of the 862 genes differentially expressed genes identified in E15.5 *Scx^-/-^* embryos, 645 (74.8%) were upregulated, therefore suggesting that similar to other bHLH proteins, Scx largely functions as a transcriptional repressor. While this could be direct repression of target DNA by Scx, this study has identified additional functions in which Scx could mediate gene regulation. Based on differential gene expression changes, processes associated with methyltransferase activity were significantly affected in the absence of *Scx* (Table 2). This includes decreases in *Dot1l* (0.66-fold) and *Mll2* (0.52-fold) which regulate methylation of histones to silence genes,[17]–[19] [20] therefore fitting with 645 genes increased in this study. In addition, several *miRNAs* were significantly decreased (*miR432* (0.09-fold), *miR-700* (0.1-fold), *miR-692-1* (0.35-fold)) which could also contribute to relieved post-transcriptional gene repression. However, using Panther and Target Scan software, we were unable to identify conserved seed sequences for these miRNAs in predicted target genes that were increased in *Scx^-/-^* embryos. Therefore suggesting that these Scx-dependent miRs are either acting indirectly on the increased gene set, or their decrease in expression is independent of differential gene expression findings. In contrast to decreased miRs, *miR-758, miR-134* and *miR-27b* were significantly increased and interestingly *miR-758* is predicted to bind conserved seed regions within *Col4a1*, a basement membrane collagen type that was found to be significantly decreased (0.67-fold, p = 5.29E-03) in *Scx^-/-^* embryos. In addition to changes in post transcriptional and post translational processes, genes associated with DNA binding were significantly altered and Ingenuity software predicted *Onecut1* as a potential upstream regulator of Scx (Figure 2A), a transcription factor previously shown to play roles in regulating gene expression.[21] However, further work is required to demonstrate this prediction. Our analysis also revealed significant decreases in *Dll4* (0.61-fold) and *Ncor2* (0.46-fold) associated with overrepresentation of Notch binding (p = 0.0007) and Notch signaling (p = 0.01) by pathway analysis (Table 2). While Notch is an important player in valve development and disease [22], a specific role in valve remodeling, or associations with Scx have not been made. Interestingly in data not shown, Scx was unable to increase activity of the Notch Intracellular Domain (NICD) in porcine VICs. As NICD expression has previously been reported in pVICs, we speculate that the Notch signaling pathway is indirectly regulated by Scx in this system [23]. These findings, based on RNA-seq and bioinformatics analyses have identified previously unappreciated roles for Scx in the regulation of gene expression. As previous studies have shown that valve development requires tight control of growth factors, transcription factors and ECM proteins [24], unveiling possible epigenetic events regulated by Scx could provide important new insights into disease mechanisms.

In remodeling heart valves, valve precursor cells are transitioning from a ‘primitive’ mesenchyme cell phenotype towards an activated VIC phenotype. This is characterized by loss of mesenchyme cell markers and maintenance of smooth muscle alpha-actin (α-SMA), an established marker of activated VICs [3]. As a myofibroblast-like cell, activated VICs exhibit an organized actin cytoskeleton and express focal adhesion proteins [3], [25]. As mentioned, mesenchyme cell markers are persistently expressed in valves isolated from *Scx^-/-^* embryos at E17.5, suggesting defects in VIC activation [9]. In an activated state, VICs mediate remodeling of the valve connective tissue which is tightly controlled and required for embryonic development, but in the adult, VICs are quiescent and therefore abnormal activation propagates pathogenic remodeling leading to disease [3], [26]. In this study, mesenchyme cell markers were not increased in *Scx^-/-^* embryos at E15.5 contrary to observations made in E17.5 *Scx^-/-^* embryos, and this discrepancy may be due to differences in the time points examined in our two studies. However, we do see significant changes in several genes associated with assembly and maintenance of the actin cytoskeleton at E17.5. These include *Phactr1* (0.55-fold), *Plectin* (0.66-fold), *Fap* (1.65-fold), *Actn4* (0.75-fold) (Figure 2A, B), *Parvb* (0.40-fold), in addition to mRNAs that regulate cell adhesion and migration (*Efna5*) associated with cellular assembly and organization processes (Table 2). Therefore, it is considered that Scx may play a significant role in mediating activated VIC phenotypes, which is not only essential for valve development, but a prominent landmark in the initiation of disease processes in adult valves.

Work from our lab and others has shown that Scx plays a major role in regulating ECM gene expression and organization in heart valves, tendons and cardiac fibroblasts [9], [11], [14]–[16]. However to our surprise, pathways analyses associated with connective tissue were not significantly altered in *Scx^-/-^* embryos at E15.5, although we did observe differential expression of ECM-related genes. Furthermore Tgfβ was predicted as an upstream regulator (Figure 2A) and has previously been shown to mediate Scx-dependent expression of ECM genes [11], [14], [15]. In recent work we showed that heart valves from *Scx^-/-^* mice at birth have decreased proteoglycan content [11] and studies in cardiac fibroblasts by The Czubryt group showed regulation of *Col1a2* by Scx in cardiac fibroblasts isolated from adult rats [15]. Therefore, we speculate that Scx-mediated regulation of the ECM is temporal and most important after birth in the valves and myocardium.

In summary, this study has shed light on several new role(s) for Scx in remodeling heart valves that could be applied to other connective tissue systems. In addition, we have generated a profile of protein-coding and non-protein-coding mRNAs whose expression is dependent upon Scx function. Many of these are associated with gene regulation and cellular development functions, however it is not yet clear which genes are directly, or indirectly regulated by Scx. Nonetheless, creating this transcriptome has not only provided a comprehensive list of mRNAs expressed in healthy remodeling heart valves (*Scx^+/+^*), but given direction for future studies identifying how defects during embryonic development cause valve disease after birth or later in life.

## Supporting Information

Table S1 **Differentially expressed non-protein coding mRNAs (<1.5-fold change, p<0.05) in** ***Scx^-/-^*** **atrioventricular canal samples compared to** ***Scx^+/+^*** **controls.**
(DOCX)

Table S2 **Exon-level alternative splicing in atrioventricular canal regions isolated from E15.5** ***Scx^-/-^*** **vs.** ***Scx^+/+^*** **embryos.**
(DOCX)
