# Supplementary material for: RNA-Seq Analysis to Identify Novel Roles of Scleraxis during Embryonic Mouse Heart Valve Remodeling
Source: PLoS One. 2014 Jul 1;9(7):e101425. doi: 10.1371/journal.pone.0101425 (PMC4077804; doi:10.1371/journal.pone.0101425)
Supplement: Table S1 — Differentially expressed non-protein coding mRNAs (<1.5-fold change, p<0.05) in Scx-/- atrioventricular canal samples compared to Scx+/+ controls. (DOCX) [file pone.0101425.s001.docx]

| **Gene** | **Description** | **Fold Change** | **p-Value** |
| --- | --- | --- | --- |
| **Non-coding Micro RNA** | | | |
| *Mir758* | microRNA 758 | 56.98 | 2.19E-03 |
| *Mir134* | microRNA 134 | 8.39 | 1.07E-03 |
| *Mir27b* | microRNA 27b | 6.24 | 5.88E-03 |
| *Mir692-1* | microRNA 692-1 | 0.35 | 3.69E-02 |
| *Mir700* | microRNA 700 | 0.10 | 1.29E-02 |
| *Mir432* | microRNA 432 | 0.09 | 4.20E-02 |
| **Non-coding nuclear/nucleolar RNA** | | | |
| *SnoU13* | Small nucleolar RNA U13 | 37.71 | 2.55E-04 |
| *Snord29* | Small nucleolar RNA SNORD29 | 29.97 | 2.33E-02 |
| *Snord99* | Small nucleolar RNA, C/D box 99 | 29.62 | 4.85E-03 |
| *Snora32* | Small nucleolar RNA SNORA32 | 19.22 | 1.06E-03 |
| *Snord104* | Small nucleolar RNA, C/D box 104 | 16.73 | 2.90E-02 |
| *Snora42* | Small nucleolar RNA SNORA42/SNORA80 family | 7.96 | 8.92E-03 |
| *U6* | U6 spliceosomal RNA | 6.64 | 3.14E-02 |
| *Snord101* | Small nucleolar RNA SNORD101 | 6.39 | 4.72E-02 |
| *Snora32* | Small nucleolar RNA SNORA32 | 5.80 | 4.76E-02 |
| *Snord93* | Small nucleolar RNA, C/D box 93 | 5.41 | 2.62E-02 |
| *U6* | U6 spliceosomal RNA | 4.31 | 3.81E-02 |
| *Snord59* | Small nucleolar RNA SNORD59 | 3.93 | 1.41E-02 |
| *Snora9* | Small nucleolar RNA SNORA9 | 0.46 | 1.20E-02 |
| *Snord95* | Small nucleolar RNA, C/D box 95 | 0.37 | 3.67E-02 |
| *Snora25* | Small nucleolar RNA SNORA25 | 0.36 | 3.11E-03 |
| *Snora21* | Small nucleolar RNA, H/ACA box 21 | 0.24 | 1.72E-02 |
| *Snora55* | Small nucleolar RNA SNORA55 | 0.15 | 2.52E-02 |
| *Snora84* | Small nucleolar RNA SNORA84 | 0.03 | 1.12E-02 |
| *Snord32a* | Small nucleolar RNA SNORD32a | N/A | 4.90E-02 |
| **Long intergenic non-coding RNA** | | | |
| *2310010G23Rik* | RIKEN cDNA 2310010G23 gene | 6.60 | 1.04E-02 |
| *Gm14261* | predicted gene 14261 | 3.62 | 1.35E-02 |
| *Gm15506* | predicted gene 15506 | 3.41 | 2.19E-02 |
| *Gm17246* | predicted gene, 17246 | 2.26 | 1.78E-02 |
| *C630043F03Rik* | RIKEN cDNA C630043F03 gene | 2.25 | 2.98E-02 |
| *Gm16765* | predicted gene, 16765 | 0.42 | 1.44E-02 |
| *Gm17639* | predicted gene, 17639 | 0.41 | 4.23E-02 |
| *A930012L18Rik* | RIKEN cDNA A930012L18 gene | 0.37 | 4.21E-02 |
| *4930513N10Rik* | RIKEN cDNA 4930513N10 gene | 0.35 | 6.56E-03 |
| *Gm10524* | predicted gene 10524 | 0.22 | 2.52E-02 |
| **Mitochondrial RNA** | | | |
| *mt-Tv* | mitochondrially encoded tRNA valine | 0.36 | 9.78E-03 |
