# Supplementary material for: RNA-Seq Analysis to Identify Novel Roles of Scleraxis during Embryonic Mouse Heart Valve Remodeling
Source: PLoS One. 2014 Jul 1;9(7):e101425. doi: 10.1371/journal.pone.0101425 (PMC4077804; doi:10.1371/journal.pone.0101425)
Supplement: Table S2 — Exon-level alternative splicing in atrioventricular canal regions isolated from E15.5 Scx-/- vs. Scx+/+ embryos. (DOCX) [file pone.0101425.s002.docx]

| **Ensembl Gene ID** | **Chromosome Name** | **Gene Start (bp)** | **Gene End (bp)** | **Strand** | **Ensembl Exon ID** | **Exon Start (bp)** | **Exon End (bp)** | **Exon Rank** | **Gene Name** | **Description** | **Fold Change** | **P Value** |
| --- | --- | --- | --- | --- | --- | --- | --- | --- | --- | --- | --- | --- |
| ENSMUSG00000019769 | 10 | 4,795,849 | 5,326,338 | 1 | ENSMUSE00000616704 | 5,111,849 | 5,111,974 | 73 | Syne1 | synaptic nuclear envelope 1 | 0.01 | 1.24E-06 |
| ENSMUSG00000030538 | 7 | 87,372,042 | 87,377,699 | -1 | ENSMUSE00000955288 | 87,372,880 | 87,372,968 | 4 | Cib1 | calcium and integrin binding 1 (calmyrin) | 37.14 | 2.04E-06 |
| ENSMUSG00000030538 | 7 | 87,372,042 | 87,377,699 | -1 | ENSMUSE00000948063 | 87,372,880 | 87,372,968 | 5 | Cib1 | calcium and integrin binding 1 (calmyrin) | 37.14 | 2.04E-06 |
| ENSMUSG00000025878 | 13 | 55,129,241 | 55,201,661 | -1 | ENSMUSE00000570937 | 55,130,777 | 55,130,819 | 12 | Uimc1 | ubiquitin interaction motif containing 1 | 72.89 | 2.21E-06 |
| ENSMUSG00000025878 | 13 | 55,129,241 | 55,201,661 | -1 | ENSMUSE00000615251 | 55,130,777 | 55,130,819 | 13 | Uimc1 | ubiquitin interaction motif containing 1 | 72.89 | 2.21E-06 |
| ENSMUSG00000030231 | 6 | 140,372,620 | 140,544,804 | 1 | ENSMUSE00000608592 | 140,375,019 | 140,375,076 | 3 | Plekha5 | pleckstrin homology domain containing, family A member 5 | 110.96 | 3.34E-06 |
| ENSMUSG00000030231 | 6 | 140,372,620 | 140,544,804 | 1 | ENSMUSE00000689308 | 140,375,019 | 140,375,076 | 3 | Plekha5 | pleckstrin homology domain containing, family A member 5 | 110.96 | 3.34E-06 |
| ENSMUSG00000025036 | 19 | 46,647,855 | 46,671,388 | 1 | ENSMUSE00000478043 | 46,647,855 | 46,647,949 | 1 | Sfxn2 | sideroflexin 2 | 0.05 | 6.13E-06 |
| ENSMUSG00000002845 | 16 | 38,558,811 | 38,592,275 | 1 | ENSMUSE00000910177 | 38,591,025 | 38,591,055 | 4 | Tmem39a | transmembrane protein 39a | 29.89 | 6.42E-06 |
| ENSMUSG00000047446 | 12 | 40,732,034 | 40,764,612 | -1 | ENSMUSE00000390764 | 40,763,850 | 40,763,954 | 1 | Arl4a | ADP-ribosylation factor-like 4A | 26.01 | 7.09E-06 |
| ENSMUSG00000014592 | 4 | 150,433,633 | 151,235,985 | -1 | ENSMUSE00000385717 | 150,537,925 | 150,538,078 | 4 | Camta1 | calmodulin binding transcription activator 1 | 0.11 | 9.18E-06 |
| ENSMUSG00000014592 | 4 | 150,433,633 | 151,235,985 | -1 | ENSMUSE00000630212 | 150,537,925 | 150,538,078 | 2 | Camta1 | calmodulin binding transcription activator 1 | 0.11 | 9.18E-06 |
| ENSMUSG00000028608 | 4 | 107,562,418 | 107,571,989 | 1 | ENSMUSE00000708843 | 107,562,474 | 107,562,501 | 1 | 0610037L13Rik | RIKEN cDNA 0610037L13 gene | 715.64 | 9.33E-06 |
| ENSMUSG00000026277 | 1 | 95,518,479 | 95,532,299 | -1 | ENSMUSE00000792492 | 95,522,628 | 95,523,710 | 5 | Stk25 | serine/threonine kinase 25 (yeast) | 1.40 | 9.91E-06 |
| ENSMUSG00000028608 | 4 | 107,562,418 | 107,571,989 | 1 | ENSMUSE00000712754 | 107,562,471 | 107,562,501 | 1 | 0610037L13Rik | RIKEN cDNA 0610037L13 gene | 647.90 | 1.00E-05 |
| ENSMUSG00000028608 | 4 | 107,562,418 | 107,571,989 | 1 | ENSMUSE00000670955 | 107,562,468 | 107,562,501 | 1 | 0610037L13Rik | RIKEN cDNA 0610037L13 gene | 590.73 | 1.06E-05 |
| ENSMUSG00000028608 | 4 | 107,562,418 | 107,571,989 | 1 | ENSMUSE00000763432 | 107,562,454 | 107,562,501 | 1 | 0610037L13Rik | RIKEN cDNA 0610037L13 gene | 420.39 | 1.35E-05 |
| ENSMUSG00000049488 | 4 | 11,966,502 | 12,017,167 | -1 | ENSMUSE00000397784 | 12,013,059 | 12,013,147 | 2 | Tmem67 | transmembrane protein 67 | 0.03 | 1.36E-05 |
| ENSMUSG00000049488 | 4 | 11,966,502 | 12,017,167 | -1 | ENSMUSE00000828354 | 12,013,059 | 12,013,147 | 2 | Tmem67 | transmembrane protein 67 | 0.03 | 1.36E-05 |
| ENSMUSG00000047446 | 12 | 40,732,034 | 40,764,612 | -1 | ENSMUSE00000655193 | 40,763,850 | 40,763,963 | 1 | Arl4a | ADP-ribosylation factor-like 4A | 24.69 | 1.44E-05 |
| ENSMUSG00000028132 | 3 | 120,904,679 | 120,986,016 | -1 | ENSMUSE00000375543 | 120,937,961 | 120,938,165 | 3 | Tmem56 | transmembrane protein 56 | 11.25 | 1.48E-05 |
| ENSMUSG00000037426 | 5 | 33,206,350 | 33,336,882 | 1 | ENSMUSE00000548614 | 33,301,930 | 33,301,995 | 10 | Depdc5 | DEP domain containing 5 | 34.04 | 1.65E-05 |
| ENSMUSG00000002205 | 7 | 52,003,783 | 52,032,885 | 1 | ENSMUSE00000829707 | 52,007,483 | 52,007,546 | 1 | Vrk3 | vaccinia related kinase 3 | 30.49 | 1.69E-05 |
| ENSMUSG00000039901 | 19 | 45,892,634 | 46,072,978 | -1 | ENSMUSE00000618415 | 46,039,654 | 46,039,740 | 8 | 9130011E15Rik | RIKEN cDNA 9130011E15 gene | 0.02 | 1.70E-05 |
| ENSMUSG00000089832 | 7 | 28,127,152 | 28,141,038 | -1 | ENSMUSE00000201162 | 28,133,553 | 28,133,640 | 11 | Shkbp1 | Sh3kbp1 binding protein 1 | 0.04 | 1.79E-05 |
| ENSMUSG00000089832 | 7 | 28,127,152 | 28,141,038 | -1 | ENSMUSE00000819807 | 28,133,553 | 28,133,640 | 9 | Shkbp1 | Sh3kbp1 binding protein 1 | 0.04 | 1.79E-05 |
| ENSMUSG00000028608 | 4 | 107,562,418 | 107,571,989 | 1 | ENSMUSE00000805954 | 107,562,430 | 107,562,501 | 1 | 0610037L13Rik | RIKEN cDNA 0610037L13 gene | 281.57 | 1.82E-05 |
| ENSMUSG00000055150 | 7 | 6,324,068 | 6,332,201 | 1 | ENSMUSE00000538208 | 6,328,229 | 6,328,324 | 3 | Zfp78 | zinc finger protein 78 | 14.02 | 1.99E-05 |
| ENSMUSG00000028608 | 4 | 107,562,418 | 107,571,989 | 1 | ENSMUSE00000745389 | 107,562,418 | 107,562,501 | 1 | 0610037L13Rik | RIKEN cDNA 0610037L13 gene | 241.34 | 2.03E-05 |
| ENSMUSG00000027546 | 2 | 168,459,938 | 168,567,909 | -1 | ENSMUSE00000170422 | 168,501,642 | 168,501,718 | 10 | Atp9a | ATPase, class II, type 9A | 65.71 | 2.04E-05 |
| ENSMUSG00000027546 | 2 | 168,459,938 | 168,567,909 | -1 | ENSMUSE00000679297 | 168,501,642 | 168,501,718 | 10 | Atp9a | ATPase, class II, type 9A | 65.71 | 2.04E-05 |
| ENSMUSG00000037270 | 3 | 36,762,028 | 36,951,955 | 1 | ENSMUSE00000675736 | 36,855,561 | 36,855,688 | 32 | 4932438A13Rik | RIKEN cDNA 4932438A13 gene | 2.48 | 2.10E-05 |
| ENSMUSG00000002205 | 7 | 52,003,783 | 52,032,885 | 1 | ENSMUSE00000274563 | 52,007,485 | 52,007,546 | 1 | Vrk3 | vaccinia related kinase 3 | 31.06 | 2.12E-05 |
| ENSMUSG00000026921 | 2 | 26,435,534 | 26,448,640 | 1 | ENSMUSE00000932081 | 26,444,063 | 26,444,081 | 1 | Egfl7 | EGF-like domain 7 | 20.35 | 2.16E-05 |
| ENSMUSG00000047446 | 12 | 40,732,034 | 40,764,612 | -1 | ENSMUSE00000736885 | 40,763,850 | 40,764,016 | 1 | Arl4a | ADP-ribosylation factor-like 4A | 16.86 | 2.39E-05 |
| ENSMUSG00000026409 | 1 | 132,585,620 | 132,625,830 | -1 | ENSMUSE00000253208 | 132,595,765 | 132,595,827 | 11 | Pfkfb2 | 6-phosphofructo-2-kinase/fructose-2,6-biphosphatase 2 | 0.02 | 2.41E-05 |
| ENSMUSG00000029446 | 5 | 130,271,434 | 130,293,129 | -1 | ENSMUSE00000809675 | 130,276,576 | 130,276,616 | 5 | Psph | phosphoserine phosphatase | 0.34 | 2.42E-05 |
| ENSMUSG00000035696 | 4 | 44,139,082 | 44,246,661 | -1 | ENSMUSE00000731178 | 44,171,845 | 44,171,924 | 2 | Rnf38 | ring finger protein 38 | 0.05 | 2.66E-05 |
| ENSMUSG00000031161 | X | 7,507,246 | 7,525,015 | -1 | ENSMUSE00000787174 | 7,508,485 | 7,508,507 | 26 | Hdac6 | histone deacetylase 6 | 20.17 | 2.93E-05 |
| ENSMUSG00000033192 | 8 | 95,379,249 | 95,443,178 | 1 | ENSMUSE00000787847 | 95,438,142 | 95,438,277 | 5 | Lpcat2 | lysophosphatidylcholine acyltransferase 2 | 0.02 | 2.99E-05 |
| ENSMUSG00000033192 | 8 | 95,379,249 | 95,443,178 | 1 | ENSMUSE00000516825 | 95,438,142 | 95,438,277 | 11 | Lpcat2 | lysophosphatidylcholine acyltransferase 2 | 0.02 | 2.99E-05 |
| ENSMUSG00000018166 | 10 | 128,004,579 | 128,026,708 | -1 | ENSMUSE00000150131 | 128,009,490 | 128,009,645 | 21 | Erbb3 | v-erb-b2 erythroblastic leukemia viral oncogene homolog 3 (avian) | 21.25 | 3.21E-05 |
| ENSMUSG00000025314 | 2 | 90,269,911 | 90,420,804 | -1 | ENSMUSE00000351978 | 90,288,216 | 90,288,306 | 16 | Ptprj | protein tyrosine phosphatase, receptor type, J | 33.63 | 3.32E-05 |
| ENSMUSG00000054509 | 14 | 57,194,456 | 57,278,631 | 1 | ENSMUSE00000864645 | 57,194,456 | 57,194,567 | 1 | Parp4 | poly (ADP-ribose) polymerase family, member 4 | 1.93 | 3.39E-05 |
| ENSMUSG00000074166 | 7 | 48,734,250 | 48,755,260 | -1 | ENSMUSE00000635212 | 48,738,203 | 48,738,329 | 1 | AW146154 | expressed sequence AW146154 | 29.42 | 3.64E-05 |
| ENSMUSG00000032436 | 9 | 114,665,955 | 114,690,963 | -1 | ENSMUSE00000689017 | 114,667,923 | 114,668,004 | 3 | Cmtm7 | CKLF-like MARVEL transmembrane domain containing 7 | 1.41 | 3.72E-05 |
| ENSMUSG00000032436 | 9 | 114,665,955 | 114,690,963 | -1 | ENSMUSE00000407626 | 114,667,923 | 114,668,004 | 4 | Cmtm7 | CKLF-like MARVEL transmembrane domain containing 7 | 1.41 | 3.72E-05 |
| ENSMUSG00000033543 | 9 | 69,860,357 | 69,870,673 | 1 | ENSMUSE00000812495 | 69,863,619 | 69,863,744 | 1 | Gtf2a2 | general transcription factor II A, 2 | 32.13 | 3.99E-05 |
| ENSMUSG00000031377 | X | 160,630,774 | 160,696,125 | -1 | ENSMUSE00000208784 | 160,684,068 | 160,684,149 | 4 | Bmx | BMX non-receptor tyrosine kinase | 32.28 | 4.34E-05 |
| ENSMUSG00000032041 | 9 | 34,992,136 | 35,007,876 | -1 | ENSMUSE00000701789 | 34,999,159 | 34,999,281 | 2 | Tirap | toll-interleukin 1 receptor (TIR) domain-containing adaptor protein | 24.21 | 4.39E-05 |
| ENSMUSG00000005672 | 5 | 75,970,941 | 76,052,747 | 1 | ENSMUSE00000187194 | 76,037,135 | 76,037,239 | 12 | Kit | kit oncogene | 29.40 | 4.49E-05 |
| ENSMUSG00000019578 | 17 | 56,206,468 | 56,214,451 | -1 | ENSMUSE00000784589 | 56,210,311 | 56,210,408 | 2 | Ubxn6 | UBX domain protein 6 | 1.59 | 4.49E-05 |
| ENSMUSG00000019578 | 17 | 56,206,468 | 56,214,451 | -1 | ENSMUSE00000139361 | 56,210,311 | 56,210,408 | 5 | Ubxn6 | UBX domain protein 6 | 1.59 | 4.49E-05 |
| ENSMUSG00000089672 | 10 | 51,200,438 | 51,206,122 | 1 | ENSMUSE00000494258 | 51,201,373 | 51,201,675 | 4 | Gp49a | glycoprotein 49 A | 0.07 | 4.70E-05 |
| ENSMUSG00000089672 | 10 | 51,200,438 | 51,206,122 | 1 | ENSMUSE00000780435 | 51,201,373 | 51,201,675 | 3 | Gp49a | glycoprotein 49 A | 0.07 | 4.70E-05 |
| ENSMUSG00000056832 | 6 | 38,331,469 | 38,377,647 | 1 | ENSMUSE00000655535 | 38,335,433 | 38,335,525 | 3 | Ttc26 | tetratricopeptide repeat domain 26 | 38.61 | 4.74E-05 |
| ENSMUSG00000022553 | 15 | 76,181,724 | 76,184,810 | 1 | ENSMUSE00000848497 | 76,182,426 | 76,182,471 | 2 | Maf1 | MAF1 homolog (S. cerevisiae) | 55.66 | 4.75E-05 |
| ENSMUSG00000075254 | 16 | 33,684,552 | 33,771,662 | 1 | ENSMUSE00000799313 | 33,756,609 | 33,756,854 | 1 | Heg1 | HEG homolog 1 (zebrafish) | 5.83 | 4.88E-05 |
| ENSMUSG00000037426 | 5 | 33,206,350 | 33,336,882 | 1 | ENSMUSE00000246369 | 33,280,794 | 33,280,954 | 4 | Depdc5 | DEP domain containing 5 | 2.41 | 5.06E-05 |
| ENSMUSG00000033068 | 2 | 150,574,778 | 150,597,411 | 1 | ENSMUSE00000816909 | 150,574,778 | 150,574,969 | 1 | Entpd6 | ectonucleoside triphosphate diphosphohydrolase 6 | 0.06 | 5.09E-05 |
| ENSMUSG00000022390 | 15 | 81,575,278 | 81,626,699 | 1 | ENSMUSE00000507748 | 81,609,040 | 81,609,139 | 11 | Zc3h7b | zinc finger CCCH type containing 7B | 0.05 | 5.36E-05 |
| ENSMUSG00000034981 | 5 | 91,946,641 | 92,055,920 | 1 | ENSMUSE00000323624 | 92,042,024 | 92,042,102 | 3 | Parm1 | prostate androgen-regulated mucin-like protein 1 | 0.02 | 5.39E-05 |
| ENSMUSG00000038122 | 10 | 55,734,104 | 55,948,495 | -1 | ENSMUSE00000905583 | 55,890,650 | 55,890,777 | 15 | D630037F22Rik | RIKEN cDNA D630037F22 gene | 0.03 | 5.55E-05 |
| ENSMUSG00000027635 | 2 | 156,821,001 | 156,832,890 | -1 | ENSMUSE00000171366 | 156,827,419 | 156,827,491 | 5 | Dsn1 | DSN1, MIND kinetochore complex component, homolog (S. cerevisiae) | 53.59 | 5.67E-05 |
| ENSMUSG00000028826 | 4 | 134,358,674 | 134,409,260 | -1 | ENSMUSE00000751404 | 134,392,682 | 134,392,949 | 3 | Tmem57 | transmembrane protein 57 | 0.06 | 5.74E-05 |
| ENSMUSG00000042473 | X | 136,219,535 | 136,287,944 | 1 | ENSMUSE00000502884 | 136,266,797 | 136,266,914 | 11 | Tbc1d8b | TBC1 domain family, member 8B | 38.88 | 5.91E-05 |
| ENSMUSG00000028024 | 3 | 128,972,093 | 129,035,638 | -1 | ENSMUSE00000175291 | 128,983,603 | 128,983,707 | 16 | Enpep | glutamyl aminopeptidase | 37.55 | 5.99E-05 |
| ENSMUSG00000042289 | 7 | 134,929,122 | 134,947,316 | 1 | ENSMUSE00000844570 | 134,942,605 | 134,942,742 | 12 | Hsd3b7 | hydroxy-delta-5-steroid dehydrogenase, 3 beta- and steroid delta-isomerase 7 | 0.05 | 6.01E-05 |
| ENSMUSG00000027635 | 2 | 156,821,001 | 156,832,890 | -1 | ENSMUSE00000804096 | 156,827,421 | 156,827,491 | 2 | Dsn1 | DSN1, MIND kinetochore complex component, homolog (S. cerevisiae) | 54.46 | 6.24E-05 |
| ENSMUSG00000028059 | 3 | 88,411,376 | 88,451,974 | 1 | ENSMUSE00000950329 | 88,419,289 | 88,419,443 | 1 | Arhgef2 | rho/rac guanine nucleotide exchange factor (GEF) 2 | 10.44 | 6.50E-05 |
| ENSMUSG00000005251 | 16 | 97,963,540 | 97,985,344 | -1 | ENSMUSE00000696861 | 97,976,675 | 97,976,966 | 2 | Ripk4 | receptor-interacting serine-threonine kinase 4 | 0.10 | 6.57E-05 |
| ENSMUSG00000005251 | 16 | 97,963,540 | 97,985,344 | -1 | ENSMUSE00000131994 | 97,976,675 | 97,976,966 | 2 | Ripk4 | receptor-interacting serine-threonine kinase 4 | 0.10 | 6.57E-05 |
| ENSMUSG00000031783 | 8 | 97,381,350 | 97,397,435 | 1 | ENSMUSE00000212934 | 97,381,647 | 97,381,696 | 2 | Polr2c | polymerase (RNA) II (DNA directed) polypeptide C | 20.17 | 6.61E-05 |
| ENSMUSG00000036591 | 2 | 20,769,546 | 20,890,508 | -1 | ENSMUSE00000928367 | 20,781,508 | 20,781,508 | 1 | Arhgap21 | Rho GTPase activating protein 21 | 323.81 | 6.77E-05 |
| ENSMUSG00000028256 | 3 | 144,781,553 | 144,816,879 | 1 | ENSMUSE00000902917 | 144,781,553 | 144,781,746 | 1 | Odf2l | outer dense fiber of sperm tails 2-like | 2.59 | 6.84E-05 |
| ENSMUSG00000026873 | 2 | 34,749,277 | 34,769,546 | -1 | ENSMUSE00000163738 | 34,752,577 | 34,752,672 | 14 | Phf19 | PHD finger protein 19 | 0.12 | 6.86E-05 |
| ENSMUSG00000026873 | 2 | 34,749,277 | 34,769,546 | -1 | ENSMUSE00000745613 | 34,752,577 | 34,752,672 | 7 | Phf19 | PHD finger protein 19 | 0.12 | 6.86E-05 |
| ENSMUSG00000041570 | 1 | 138,164,700 | 138,242,681 | -1 | ENSMUSE00000690276 | 138,190,587 | 138,190,623 | 6 | Camsap2 | calmodulin regulated spectrin-associated protein family, member 2 | 70.15 | 7.89E-05 |
| ENSMUSG00000021645 | 13 | 100,894,807 | 100,907,645 | 1 | ENSMUSE00000903432 | 100,896,825 | 100,896,873 | 3 | Smn1 | survival motor neuron 1 | 53.23 | 7.99E-05 |
| ENSMUSG00000018395 | 11 | 53,380,881 | 53,417,746 | 1 | ENSMUSE00000831725 | 53,392,617 | 53,392,686 | 1 | Kif3a | kinesin family member 3A | 28.22 | 8.08E-05 |
| ENSMUSG00000000301 | 11 | 59,784,116 | 59,859,991 | -1 | ENSMUSE00000590127 | 59,784,116 | 59,784,377 | 3 | Pemt | phosphatidylethanolamine N-methyltransferase | 0.30 | 8.20E-05 |
| ENSMUSG00000000301 | 11 | 59,784,116 | 59,859,991 | -1 | ENSMUSE00000662312 | 59,784,116 | 59,784,377 | 7 | Pemt | phosphatidylethanolamine N-methyltransferase | 0.30 | 8.20E-05 |
| ENSMUSG00000024112 | 17 | 25,511,230 | 25,570,728 | -1 | ENSMUSE00000611168 | 25,521,212 | 25,521,396 | 20 | Cacna1h | calcium channel, voltage-dependent, T type, alpha 1H subunit | 0.09 | 8.81E-05 |
| ENSMUSG00000005672 | 5 | 75,970,941 | 76,052,747 | 1 | ENSMUSE00000739582 | 76,037,108 | 76,037,239 | 1 | Kit | kit oncogene | 25.00 | 8.83E-05 |
| ENSMUSG00000006585 | 8 | 125,091,915 | 125,097,028 | 1 | ENSMUSE00000215091 | 125,095,596 | 125,095,748 | 8 | Cdt1 | chromatin licensing and DNA replication factor 1 | 8.73 | 9.02E-05 |
| ENSMUSG00000037335 | 11 | 57,642,207 | 57,646,320 | -1 | ENSMUSE00000859987 | 57,644,746 | 57,645,458 | 2 | Hand1 | heart and neural crest derivatives expressed transcript 1 | 5.42 | 9.13E-05 |
| ENSMUSG00000018395 | 11 | 53,380,881 | 53,417,746 | 1 | ENSMUSE00000945481 | 53,392,602 | 53,392,686 | 4 | Kif3a | kinesin family member 3A | 31.25 | 9.17E-05 |
| ENSMUSG00000018395 | 11 | 53,380,881 | 53,417,746 | 1 | ENSMUSE00000678613 | 53,392,602 | 53,392,686 | 4 | Kif3a | kinesin family member 3A | 31.25 | 9.17E-05 |
| ENSMUSG00000047617 | 2 | 25,310,661 | 25,316,614 | -1 | ENSMUSE00000759478 | 25,315,687 | 25,315,799 | 1 | BC029214 | cDNA sequence BC029214 | 20.94 | 9.19E-05 |
| ENSMUSG00000033991 | 13 | 76,236,182 | 76,327,764 | 1 | ENSMUSE00000328989 | 76,280,631 | 76,280,724 | 9 | Ttc37 | tetratricopeptide repeat domain 37 | 76.55 | 9.19E-05 |
| ENSMUSG00000028256 | 3 | 144,781,553 | 144,816,879 | 1 | ENSMUSE00000635414 | 144,781,564 | 144,781,746 | 1 | Odf2l | outer dense fiber of sperm tails 2-like | 2.61 | 9.64E-05 |
| ENSMUSG00000064120 | 17 | 49,567,687 | 49,594,760 | 1 | ENSMUSE00000933129 | 49,593,355 | 49,593,526 | 3 | Mocs1 | molybdenum cofactor synthesis 1 | 10.24 | 9.66E-05 |
| ENSMUSG00000024845 | 19 | 4,125,934 | 4,132,307 | 1 | ENSMUSE00000843723 | 4,127,698 | 4,127,739 | 3 | Tmem134 | transmembrane protein 134 | 19.68 | 9.69E-05 |
| ENSMUSG00000015363 | 15 | 88,905,554 | 88,917,507 | 1 | ENSMUSE00000900526 | 88,912,959 | 88,915,876 | 1 | Trabd | TraB domain containing | 1.36 | 9.73E-05 |
| ENSMUSG00000028800 | 4 | 129,193,348 | 129,219,957 | -1 | ENSMUSE00000182298 | 129,196,296 | 129,196,404 | 8 | Hdac1 | histone deacetylase 1 | 0.24 | 9.74E-05 |
| ENSMUSG00000028800 | 4 | 129,193,348 | 129,219,957 | -1 | ENSMUSE00000778670 | 129,196,296 | 129,196,404 | 3 | Hdac1 | histone deacetylase 1 | 0.24 | 9.74E-05 |
| ENSMUSG00000039474 | 5 | 37,357,343 | 37,380,221 | -1 | ENSMUSE00000285203 | 37,362,797 | 37,362,948 | 7 | Wfs1 | Wolfram syndrome 1 homolog (human) | 17.12 | 9.78E-05 |
| ENSMUSG00000031641 | 8 | 63,966,531 | 63,982,279 | 1 | ENSMUSE00000395545 | 63,981,830 | 63,982,279 | 5 | Cbr4 | carbonyl reductase 4 | 1.63 | 9.81E-05 |
| ENSMUSG00000060261 | 5 | 134,713,704 | 134,790,630 | -1 | ENSMUSE00000938251 | 134,719,377 | 134,719,469 | 26 | Gtf2i | general transcription factor II I | 0.56 | 1.01E-04 |
| ENSMUSG00000026946 | 2 | 51,804,007 | 51,829,014 | -1 | ENSMUSE00000841603 | 51,828,108 | 51,828,147 | 2 | Nmi | N-myc (and STAT) interactor | 82.42 | 1.01E-04 |
